# The Prediction of Running Velocity during the 30–15 Intermittent Fitness Test Using Accelerometry-Derived Metrics and Physiological Parameters: A Machine Learning Approach

**DOI:** 10.3390/ijerph182010854

**Published:** 2021-10-15

**Authors:** Andrea Di Credico, David Perpetuini, Piero Chiacchiaretta, Daniela Cardone, Chiara Filippini, Giulia Gaggi, Arcangelo Merla, Barbara Ghinassi, Angela Di Baldassarre, Pascal Izzicupo

**Affiliations:** 1 Department of Medicine and Aging Sciences, University “G. d’Annunzio” of Chieti-Pescara, 66100 Chieti, Italy; andrea.dicredico@unich.it (A.D.C.); giulia.gaggi@unich.it (G.G.); b.ghinassi@unich.it (B.G.); izzicupo@unich.it (P.I.); 2 Department of Neurosciences, Imaging and Clinical Sciences, University “G. d’Annunzio” of Chieti-Pescara, 66100 Chieti, Italy; david.perpetuini@unich.it (D.P.); d.cardone@unich.it (D.C.); chiara.filippini@unich.it (C.F.); arcangelo.merla@unich.it (A.M.); 3 Department of Psychological, Health and Territory Sciences, University “G. d’Annunzio” of Chieti-Pescara, 66100 Chieti, Italy; p.chiacchiaretta@unich.it; 4 Center for Advanced Studies and Technology (CAST), University “G. d’Annunzio” of Chieti-Pescara, 66100 Chieti, Italy

**Keywords:** training load, physiology, HIIT, heart rate, acceleration, support vector machine, global positioning system, inertial measurement unit

## Abstract

Measuring exercise variables is one of the most important points to consider to maximize physiological adaptations. High-intensity interval training (HIIT) is a useful method to improve both cardiovascular and neuromuscular performance. The 30–15_IFT_ is a field test reflecting the effort elicited by HIIT, and the final velocity reached in the test is used to set the intensity of HIIT during the training session. In order to have a valid measure of the velocity during training, devices such as GPS can be used. However, in several situations (e.g., indoor setting), such devices do not provide reliable measures. The aim of the study was to predict exact running velocity during the 30–15_IFT_ using accelerometry-derived metrics (i.e., Player Load and Average Net Force) and heart rate (HR) through a machine learning (ML) approach (i.e., Support Vector Machine) with a leave-one-subject-out cross-validation. The SVM approach showed the highest performance to predict running velocity (r = 0.91) when compared to univariate approaches using *PL* (r = 0.62), AvNetForce (r = 0.73) and HR only (r = 0.87). In conclusion, the presented multivariate ML approach is able to predict running velocity better than univariate ones, and the model is generalizable across subjects.

## 1. Introduction

Measuring the training load consists of recording physiological and psychological requirements during exercise training and competition periods in order to maximize training adaptation and minimize overtraining and the injury risk [1,2,3]. Training load is a construct comprising two components: the external and the internal training load. External training load represents the physical workload imposed on the subject (e.g., distance covered in a run, weight lifted), while internal training load denotes the physiological, psychological and biomechanical responses of the subject to the imposed stimuli (i.e., to the external training load) [4,5,6,7,8]. External training load is typically measured using micro-sensors and time-motion analysis [9,10,11], while internal training load can be assessed using physiological indices including heart rate (HR), oxygen consumption, lactate concentration and rating of perceived exertion (RPE) [12,13,14,15,16]. One type of training in which assessing training load and regulate exercise variables accordingly are key practices is high-intensity interval training (HIIT) [17]. HIIT includes repeated bouts of high-intensity exercise interspersed with recovery periods, where high intensity generally means spending several minutes of the exercise session at least at 90% of maximal oxygen uptake ($\dot{V}$O_2max_) [17,18], and reflects the model of physical effort experienced in team sports training sessions (e.g., ice hockey, basketball, rugby and soccer), thus representing a useful tool for enhancing performance in team sport athletes [19,20,21,22]. Since HIIT protocols elicit $\dot{V}$O_2max_, they maximally involve oxygen transport and consumption and stimulate specific signaling pathways, providing an optimal stimulus to increase cardiorespiratory capacity and thus endurance performance [23,24,25,26]. The two intermittent fitness tests used to prescribe exercise intensity for HIIT are the Yo-Yo Intermittent Recovery Test (Yo-Yo IR) [27] and the 30–15 Intermittent Fitness Test (30–15_IFT_) [28]. The 30–15_IFT_ is a field test specifically developed to resolve training intensity prescription for HIIT. In addition to cardiorespiratory fitness, the 30–15_IFT_ was designed to assess anaerobic capacity, inter-effort recovery abilities and the change in direction ability [28], with all these parameters fundamental in high-intensity intermittent efforts. The final velocity reached in the 30–15_IFT_ (i.e., the V_IFT_) is used to prescribe the training intensity for HIIT. Nevertheless, it was suggested that the final velocity reached in the Yo-Yo IR1 is not as accurate as the V_IFT_ for velocity-based exercise prescription since its relationship with $\dot{V}$O_2max_ is speed-dependent [29]. When running at vYo-Yo IR1, slow and unfit athletes would use a greater proportion of their anaerobic speed reserve, while fitter athletes would run below their $\dot{V}$O_2max_ [17]. Accordingly, obtaining precise measures of running velocity is fundamental. In this regard, accelerometry is a relatively recent method used to quantify external training load (including velocity) in team sports [30] and physical activity in different populations [31,32,33]. Triaxial accelerometers present high acquisition rates and measure the activities in three orthogonal planes of motion. On the contrary, Global Positioning Systems (GPS), which are also used to assess external training load, can measure activities only in one plane of motion and can be unreliable because of intermittent signal when insufficient satellites connection occurs [34]. They can provide good estimates of external training load (e.g., velocity, total distance, speed zones) only during outdoor physical activities [35]. Compared to GPS, accelerometers have the potential also to quantify movements such as jumping, change in direction, shuffling and concussion in both outdoor and indoor settings [36,37]. Two main metrics used to assess the external training load are the average net force (AvNetForce) and the Player Load (*PL*). AvNetForce is an accelerometry-derived metric that is indicative of objective exercise intensity and offers a valuable method to quantify the intensity during intermittent efforts. AvNetForce is obtained by multiplying the vector magnitude units (*VMU*) by the subject weight [38]. PL is a proprietary formula created by Catapult Sports, and it represents a variable used for quantifying the total workload, and it is measured in arbitrary units [39].

These two metrics were employed by Staunton et al. [37] to assess the construct validity of accelerometry-derived net force to quantify the external load during basketball movements. The external load during the basketball exercise simulation test (BEST) was estimated employing a within-player model developed considering the correlation between running speed in the Yo-Yo IR1 and the accelerometry-derived AvNetForce [37]. These findings indeed demonstrated the possibility to generalize the results obtained during the Yo-Yo IR1 for other exercises. The need to implement a within-player model instead of a between-player model highlights a variability across subjects of the correlation between running speed and accelerometry-derived metrics.

In order to overcome this issue, a machine learning (ML) approach is highly suited. ML is a field of applied statistics that, instead of inferring confidence intervals of variables of interest, employs multivariate approaches for prediction purposes [40]. Recently, in sport science, it was observed that ML approaches could be useful to evaluate both for automating sports movement recognition and physical activity intensity measured by triaxial accelerometers [41]. Indeed, different physical activity typologies, energy expenditure and intensities can be measured from raw acceleration data using machine learning approaches [42,43,44]. In a multivariate framework, it is possible to combinate external and internal training load information to obtain an estimate of an unknown parameter (e.g., the velocity of running in a training session). Obtaining accurate values of exercise intensity, such as velocity, is of key importance given that the adaptations of the human body are highly specific to the typology of imposed demand.

Since the V_IFT_ can be used to prescribe the training intensity for HIIT, obtain precise velocities during this test in different conditions (e.g., both indoor and outdoor) is of utmost importance. Thus, the aim of this study was to demonstrate the capability of a multivariate data-driven ML approach to predict the exact velocity in the 30–15_IFT_. Specifically, a support-vector machine (SVM) framework was fed with parameters indicative of both the internal (i.e., heart rate) and the external (i.e., AvNetForce and *PL*) load, evaluated in a semi-professional soccer team. In order to test the generalization capabilities of the approach, a leave-one-subject-out cross-validation was also implemented.

## 2. Materials and Methods

### 2.1. Participants

Twenty-six semi-professional soccer players (age = 19.83 ± 1.25 years) from a competitive regional-level team participated in the study. After baseline testing, 5 players were initially excluded from the sample due to injuries unrelated to the proposed testing interventions. The intervention took place during the preseason training period. Twenty-one players completed the study. The technical department of the soccer club approved the study procedures and interventions. The present data arose as a condition of regular monitoring and training manipulation defined by the investigated club. The researchers only supported the appropriate design of the data collection. Therefore, because of the a posteriori nature of the analyses without interfering in the training routine, a signature of the informed consent form was not required [45].

### 2.2. Height, Weight and BMI

All the anthropometric measurements were performed by a certified specialist (i.e., a level 1 certification of the International Society for the Advancement of Kinanthropometry (ISAK)). Subjects wore light clothing and had fasted for at least 12 h before the assessments. Height was measured to the nearest 0.1 cm, and body weight was measured to the nearest 0.1 kg using a stadiometer with a balance-beam scale (Seca 200, Seca, Hamburg, Germany). Body mass index (BMI) was calculated as weight in kilograms divided by the square of height, expressed in meters.

### 2.3. Procedures

Athletes participated in two familiarization training sessions one week before the beginning of the study. The week before and after the preseason training period, athletes were assessed for the 30–15 Intermittent Fitness Test (30–15_IFT_) performance [28]. All measurements were completed under the same standardized conditions in a grass soccer field where the athletes regularly trained, wearing habitual soccer garments and boots. In addition, participants were asked to avoid consumption of caffeine-containing beverages and alcohol on the testing days, to continue their habitual daily dietary regimen and to be well hydrated. They were also required to avoid heavy activities in the 24 h preceding the tests. A standardized warm-up consisting of 5 min jogging, 5 squat jumps, 5 countermovement jumps, and 3 × 15 m sprint was completed before pre- and post-preseason training period measurements. During the entire test session, players wore a commercial triaxial accelerometer (GT9X Link; Actigraph, Pensacola, FL, USA) inside a pouch positioned on the posterior torso at the level of the inferior angle of the scapulae [39,46]. The initialization of the ActiGraph accelerometer and chest mount Polar H7 HR monitor (Polar Electro Oy, Kempele, Finland) was performed by using the ActiLife6 software (version 6.12.1, ActiGraph, Cary, NC, USA). The sampling frequency of the accelerometer and heart rate monitor was set to 100 Hz, and HR data were collected in 1-s intervals set manually via ActiLife6 software.

### 2.4. The 30–15 Intermittent Fitness Test

The 30–15_IFT_ was conducted according to the procedures outlined by Buchheit [28]. Briefly, athletes performed 30 s shuttle runs interspersed with 15 s of walking recovery, having an initial velocity of 8 km·h^−1^ with increments of 0.5 km·h^−1^ every 45 s. The 30–15_IFT_ was performed over a 40 m shuttle distance, where the subject had to run back and forth at a pace governed by a prerecorded beep so that at each short beep sound subjects should be within 3 min of zones placed at each extremity or in the middle of the course. During the 15 s recovery period, athletes walked in the forward direction towards the closest start line, where they would begin the next stage from the standing position. Exhaustion was defined as an inability to complete the required distance before the occurrence of the audio signal on three consecutive occasions. The last completed stage was deemed to be the final velocity reached in the test (V_IFT_).

### 2.5. Preprocessing

Regarding the accelerometry data, the raw signal was corrected for high-frequency motions artifacts considering the envelope of the signal. The corrupted epochs were assessed by visual inspection and removed from the analysis. The corrected signal was divided into temporal windows corresponding to the different running speeds of 30–15_IFT._ On these temporal windows, *PL* [47] (Equation (3)) and AvNetForce were computed. In order to obtain AvNetForce, epochs of 30 sec of Instantaneous Net force were averaged (Equation (2)) (Instantaneous Net force was obtained multiplying the *VMU* by the subject’s weight (Equation (1)). The raw acceleration on the three axes (*x*, *y*, *z*) used to compute *VMU*, AvNetForce, and PL were easily obtained using the proprietary software ActiLife6 software (version 6.12.1, ActiGraph, Cary, NC, USA). Concerning the HR data, the average HR was computed for each temporal window considered (i.e., every 30 s) to match accelerometry variables. Importantly, a trimmean approach was employed in order to exclude the outliers from the analysis. This approach allowed to obtain 341 temporal windows from the 21 participants.

(1) $$VMU=\sqrt{x^{2}+y^{2}+z^{2}}$$

(2) $$Istantaneous\;Net\;Force=Subject^{\prime }s\;mass\times VMU$$

(3) $$PL=\sqrt{\frac{{\left(a_{y1}-a_{y-1}\right)}^{2}+{\left(a_{x1}-a_{x-1}\right)}^{2\;}{\left(a_{z1}-a_{z-1}\right)}^{2}}{100}}$$

### 2.6. Statistical Analysis

An in-sample correlation analysis between the running speed, the accelerometry derived metrics (i.e., AvNetForce and *PL*) and the HR was performed. Specifically, this descriptive statistical analysis was performed, including all the samples in order to investigate the relationship between the running speed and the metrics evaluated. The correlation coefficient is indicative of the strength of the relationship between two variables. Several approaches were proposed to make the correlation coefficient a descriptor of the extent of the investigated relationship by setting some thresholds. Specifically, in this work the magnitude of correlations was considered: r = 0.00–0.09, negligible; r = 0.10–0.39, weak; r = 0.40–0.69, moderate; r = 0.70–0.89, strong; and r = 0.90–1.00, very strong, in accordance with the cut-off points defined by Schober et al. [48]. Importantly, the coefficient of the linear fitting was considered to assess the variability across subjects.

Concerning the ML approach, a regression based on SVM was implemented using a linear kernel. The features used as input of the model were the HR, *PL* and AvgNetForce, whereas the output was the running velocity. Of note, the metrics were normalized (z-score). The generalization capabilities of the model were tested through a leave-one-subject-out cross-validation [49].

**Figure 1:**
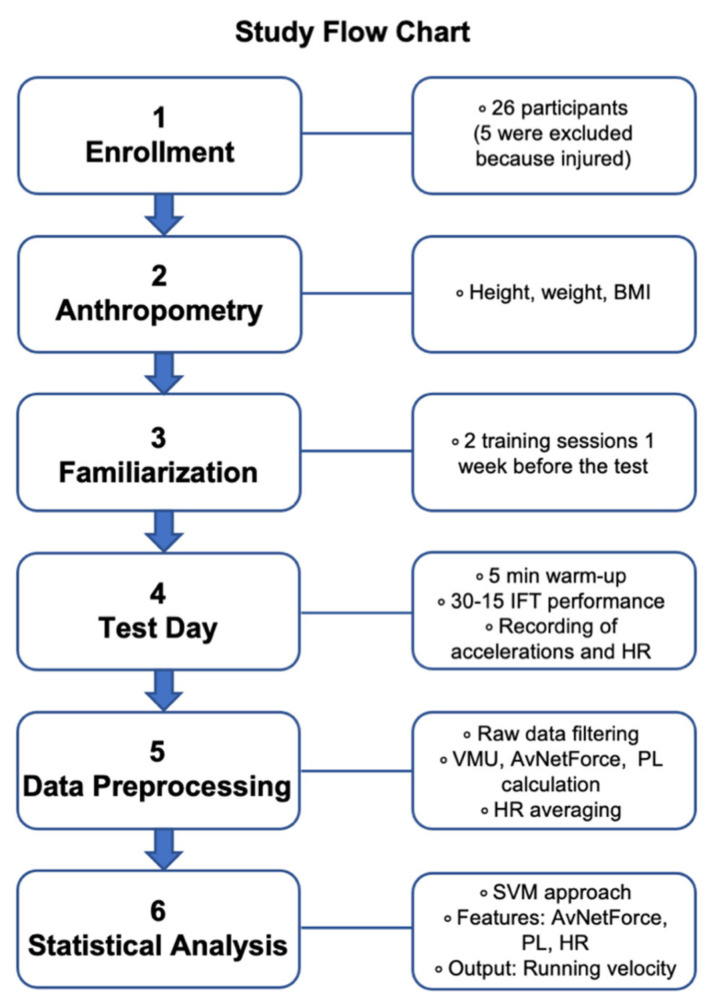
Study flow chart. It indicates the different experimental stages. BMI = body mass index; 30–15 IFT = 30–15 intermittent fitness test; HR = heart rate; *VMU* = vector magnitude units; AvNetForce = average net force; *PL* = Player Load.

Notably, the machinery was fed with 341 samples from 21 subjects; hence, all the samples from each subject were excluded during the leave-one-subject-out cross-validation. The performance of the model in estimating the running speed was evaluated by correlation analysis, Bland–Altman plot, and paired t-test. Finally, the performance of the cross-validated multivariate approach was compared to those of the out-sample univariate approaches (i.e., employing the input features considered separately) to assess the advantage of the multivariate procedure. It is worth highlighting that this approach differs from the descriptive analysis because it is evaluated by leaving out samples from one subject at a time in an iterated framework. In fact, the aim of the ML procedure is not to describe the relationship between the variables but to predict the dependent variable from the independent one. All statistical analysis were performed in MATLAB. Additionally, a schematic study flow chart is provided in Figure 1.

## 3. Results

### 3.1. Descriptive Statistics, Correlation Analysis and in-Sample Linear Regression

**Figure 2:**
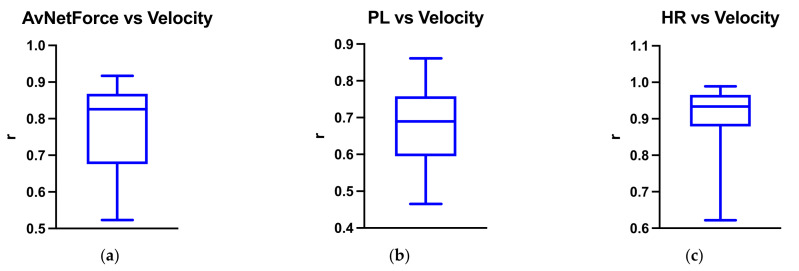
The figure reports the average and standard deviation (SD) of the correlation coefficient (r) between the velocity and the metrics considered. AvNetForce = average net force; *PL* = Player Load; HR = heart rate. (**a**) AvNetForce vs Velocity; (**b**) PL vs Velocity; (**c**) HR vs Velocity.

Table 1 reports descriptive statistics of the sample. The in-sample correlation analysis between the velocity and the metrics considered showed that PL was moderately correlated with velocity (r = 0.68). AvNetForce exhibited a strong correlation (r = 0.78), whereas HR highlighted a very strong correlation with velocity (r = 0.91). Importantly, a very low inter-subject variability of correlation coefficients between velocity and AvNetForce (Figure 2a, SD = 0.11), PL (Figure 2b, SD = 0.097), and HR (Figure 2c, SD = 0.081) was found.

**Figure 3:**
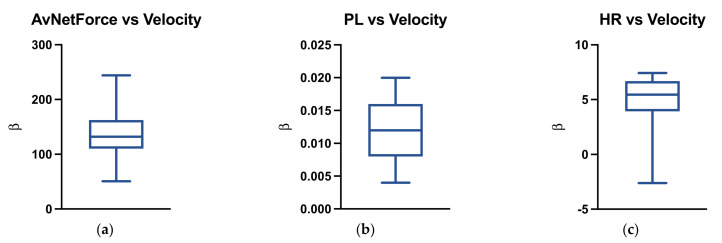
The figure reports the average and SD of the coefficient of the linear fitting (β) between velocity and the metrics considered. AvNetForce = average net force; *PL* = Player Load; HR = heart rate. (**a**) AvNetForce vs Velocity; (**b**) PL vs Velocity; (**c**) HR vs Velocity.

Moreover, the average coefficient of the linear fitting between velocity and the metrics considered were 132.46 ± 18.87 for the AvNetForce (Figure 3a), 0.012 ± 0.005 for the PL (Figure 3b) and 3.92 ± 3.68 for the HR (Figure 3c).

### 3.2. SVM and Agreement

**Figure 4:**
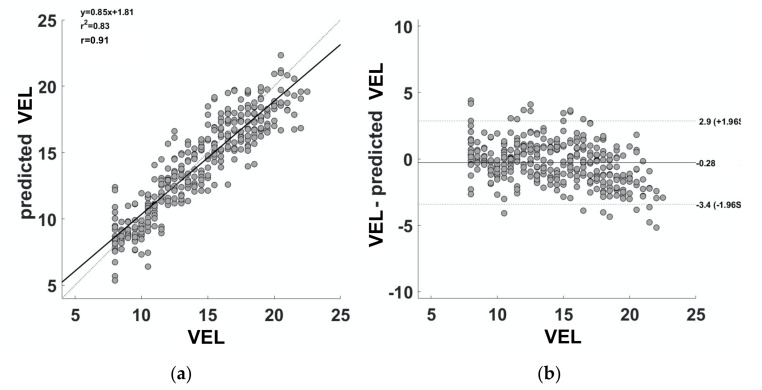
The figure reports the correlation (**a**) and the Bland-Altman (**b**) plots obtained between the estimated and the known velocities. *VEL* = velocity.

The SVM delivered a cross-validated model able to predict the velocity during the 30–15_IFT_ with a correlation coefficient of 0.91 (Figure 4a). The linear equation linking the predicted and the real velocity is:

(4) Predicted VEL=0.85×VEL+1.81

The Bland–Altman plot (Figure 4b) showed a high agreement between the velocity during the 30–15_IFT_ and the predicted velocity (mean difference = −0.28, upper LoA = 2.9; lower LoA = −3.4). In addition, a paired t-test between the velocity and the predicted velocity revealed no significant differences (t = 1.386; df = 340; *p* = 0.167).

Additionally, Table 2 reports the out-of-sample correlation coefficient of the multivariate (i.e., SVM) and univariate approaches (i.e., AvNetForce, *PL*, HR), showing a significantly higher performance of the multivariate method with respect to the univariate ones.

## 4. Discussion

In this study, an ML approach to predict running velocity using accelerometry-derived metrics (i.e., AvNetForce and *PL*) and physiological parameters (i.e., HR) measured during the execution of the 30–5_IFT_ was used_._ The SVM provided a nearly perfect correlation between predicted velocity and the running velocity during the 30–15_IFT._ In addition, the univariate approaches (using AvNetForce, *PL*, and HR separately) demonstrate lower performance.

Measuring external load is of utmost importance during training and competition. In a team sport, for example, GPS and accelerometry are the most used devices [30,36,50,51]. However, the reliability of GPS is affected by sample rate, the typology of the task and the velocity [5,35]. Indeed, it was found that the higher the velocity during a task, the lower the GPS reliability [52]. Another limitation of commercially available GPS is that it can track velocity only during outdoor activities. In this regard, there is a need to find methods to monitor parameters of external load (e.g., running velocity) during indoor activities [35]. Thus, the presented approach provides an alternative to meet such necessities.

Moreover, the predicted velocity obtained using the accelerometry-derived metrics and HR was compared with the running velocity during the 30–15_IFT_. The final velocity reached in this test could be used to prescribe the intensity of running-based HIIT. For example, for short interval running-based HIIT (e.g., with a duration of <60 s of work interval), the velocity prescribed is usually 89/105% of V_IFT_ [17,53]. On the contrary, repeated sprint training, a type of HIIT, is formed by work intervals lasting from 3 to 7 s with a velocity corresponding to 100–160% V_IFT_. Importantly, each type of HIIT leads to specific adaptations, where lower velocities with longer duration taxes predominantly metabolic (O_2_ system) and higher velocities with shorter duration involved a major neuromuscular component [53,54]. Thus, having the ability to monitor and prescribe accurate velocities during training is of main importance.

The results displayed that the in-sample analysis showed a moderate correlation between *PL* and velocity, a strong correlation between AvNetForce and velocity and a very strong between HR and velocity. However, the analysis of the coefficient of the linear fitting highlights a great variability among the subjects of the relationship between the velocity and the metrics considered, confirming the results of previous studies [37].

This finding confirms the necessity of an ML approach to estimate the velocity that could be generalizable across the subjects.

In order to investigate the generalization performance of the model, a leave-one-subject-out cross-validation approach was implemented to estimate the prediction capability of the model on a novel dataset, hence providing an unbiased estimation of the algorithm performance.

The capability of the cross-validated ML approach to estimate the velocity during the 30–15_IFT_ from accelerometry (using AvNetForce and *PL*) and physiological metrics (i.e., HR) was tested by means of a correlation analysis: Bland–Altman plot and t-test. The correlation analysis showed a strong correlation between the velocity during the 30–15_IFT_ and the predicted velocity. The Bland–Altman plot demonstrated that the errors in the estimation of velocity with respect to the real running velocity during the test were distributed within the 95% confidence interval, showing a good correspondence of the two methods without clear outliers.

However, it should be highlighted that a slight systematic error of the model in the estimation of the velocity is present (i.e., underestimation of the velocity at high values). This error is related to the fitting equation linking the predicted and real velocities. In fact, a perfect model would deliver a slope of the linear fitting equal to 1, and the line should pass through the origin of the coordinate system. In this case, the slope is lower than 1, and the *y*-axis intercept is 1.81. However, although the parameters of the linear fitting are not optimal, the paired t-test did not show a significant difference between the real and the predicted velocities, thus demonstrating good performances of the model.

Importantly, the multivariate SVM approach performed better with respect to all the univariate regressions, including the HR, which was the best-correlated variable with the velocity. Further studies should be performed to enlarge the sample size of the population. In fact, ML frameworks rely on data-driven analysis that might greatly increase their performances with large sample sizes. Furthermore, increasing the sample size allows to decrease the risk of a possible in-sample overfitting effect of the regressor and to increase the number of input regressors.

Moreover, it would be worth investigating more complex non-linear machinery such as Deep Learning [55,56] to predict the velocity from accelerometry and physiological signals in order to obtain more precise measures.

## 5. Conclusions

This is the first study aiming to predict the precise velocity during the 30–15_IFT_ using heart rate and accelerometry-derived metrics through an ML approach with a leave-one-subject-out cross-validation. The SVM provides the best performance in predicting running velocity with very high precision using the selected metrics. In addition, these results offer a practical opportunity for prescribing and monitoring velocity during running-based HIIT using accelerometry and HR where GPS is not able to provide reliable measures. Importantly, this method allows the generalization of the model to anyone, offering a further modality to monitoring performance and training adaption and prescribe exercise intensity for HIIT. Such an approach is of utmost importance also from a physiological point of view, since different physical demands elicit specific adaptation to training; thus, the more precise are the measures used to prescribe training, the better the physiological adaptation.

## Figures and Tables

**Table 1:** Descriptive statistics of the sample.

	M ± SD	Min–Max	CV (%)
Age (years)	26.72 ± 6.43	20–41	24.08
Height (cm)	177.5 ± 8.08	157–190.2	4.55
Weight (kg)	71.93 ± 11.14	48.4–94	15.49
BMI	22.71 ± 2.36	19.21–28.55	10.39
Min HR (bpm)	59.8 ± 8.53	49–79	14.26
Max HR (bpm)	197.07 ± 8.39	181–209	4.27
V_IFT_ (km/h)	19.78 ± 0.94	18.5–21.5	4.77

BMI = body mass index; V_IFT_ = final velocity reached in the 30–15 Intermittent Fitness Test.

**Table 2:** The table reports the correlation coefficients of the multivariate and univariate cross-validated approaches (*p* ~ 0) and the results of the statistical comparison between the multivariate and univariate methods.

	r	z-Statistics (SVM vs. UNIVARIATE)	*p*-Value
SVM	0.91	-	-
AvNetForce	0.73	7.78	~0
*PL*	0.62	10.43	~0
HR	0.87	2.53	0.01

SVM = Support Vector Machine; AvNetForce = average net force; *PL* = Player Load; HR = heart rate.

## Data Availability

Data are available on reasonable request from the authors.

## References

[1] Bourdon P.C., Cardinale M., Murray A., Gastin P., Kellmann M., Varley M.C., Gabbett T.J., Coutts A.J., Burgess D.J., Gregson W. (2017). Monitoring athlete training loads: Consensus statement. Int. J. Sports Physiol. Perform..

[2] Marino F.E. (2011). Physiology—laboratory and clinical research. Regulation of Fatigue in Exercise.

[3] Impellizzeri F.M., Menaspà P., Coutts A.J., Kalkhoven J., Menaspà M.J. (2020). Training load and its role in injury prevention, Part I: Back to the future. J. Athl. Train..

[4] McLaren S.J., Macpherson T.W., Coutts A.J., Hurst C., Spears I.R., Weston M. (2018). The relationships between internal and external measures of training load and intensity in team sports: A meta-analysis. Sports Med..

[5] Halson S.L. (2014). Monitoring training load to understand fatigue in athletes. Sports Med..

[6] Impellizzeri F.M., Marcora S.M., Coutts A.J. (2019). Internal and external training load: 15 years on. Int. J. Sports Physiol. Perform..

[7] Izzicupo P., D’Amico M.A., Bascelli A., Di Fonso A., D’Angelo E., Di Blasio A., Bucci I., Napolitano G., Gallina S., Di Baldassarre A. (2013). Walking training affects dehydroepiandrosterone sulfate and inflammation independent of changes in spontaneous physical activity. Menopause.

[8] Falone S., Mirabilio A., Passerini A., Izzicupo P., Cacchio M., Gallina S., Baldassarre A.D., Amicarelli F. (2009). Aerobic performance and antioxidant protection in runners. Int. J. Sports Med..

[9] Campos-Vazquez M.A., Toscano-Bendala F.J., Mora-Ferrera J.C., Suarez-Arrones L.J. (2017). Relationship between internal load indicators and changes on intermittent performance after the preseason in professional soccer players. J. Strength Cond. Res..

[10] Stiles V.H., Pearce M., Moore I.S., Langford J., Rowlands A.V. (2018). Wrist-worn accelerometry for runners: Objective quantification of training load. Med. Sci. Sports Exerc..

[11] Polglaze T., Dawson B., Hiscock D.J., Peeling P. (2015). A comparative analysis of accelerometer and time-motion data in elite men’s hockey training and competition. Int. J. Sports Physiol. Perform..

[12] Borresen J., Lambert M.I. (2009). The quantification of training load, the training response and the effect on performance. Sports Med..

[13] Haddad M., Stylianides G., Djaoui L., Dellal A., Chamari K. (2017). Session-RPE method for training load monitoring: Validity, ecological usefulness, and influencing factors. Front. Neurosci..

[14] Malone S., Hughes B., Collins K., Akubat I. (2020). Methods of monitoring training load and their association with changes across fitness measures in hurling players. J. Strength Cond. Res..

[15] Izzicupo P., Ghinassi B., D’Amico M.A., Di Blasio A., Gesi M., Napolitano G., Gallina S., Di Baldassarre A. (2013). Effects of ACE I/D polymorphism and aerobic training on the immune-endocrine network and cardiovascular parameters of postmenopausal women. J. Clin. Endocrinol. Metab..

[16] Izzicupo P., Di Valerio V., D’Amico M.A., Di Mauro M., Pennelli A., Falone S., Alberti G., Amicarelli F., Miscia S., Gallina S. (2010). Nad(P)H oxidase and pro-inflammatory response during maximal exercise: Role of C242T polymorphism of the P22PHOX subunit. Int. J. Immunopathol. Pharmacol..

[17] Buchheit M., Laursen P.B. (2013). High-intensity interval training, solutions to the programming puzzle: Part I: Cardiopulmonary emphasis. Sports Med..

[18] Billat V. (2001). Interval training for performance: A scientific and empirical practice. Special recommendations for middle- and long-distance running. Part I: Aerobic interval training. Sports Med..

[19] Iaia F.M., Bangsbo J. (2010). Speed endurance training is a powerful stimulus for physiological adaptations and performance improvements of athletes. Scand. J. Med. Sci. Sports.

[20] Kelly D.T., Tobin C., Egan B., McCarren A., O’Connor P.L., McCaffrey N., Moyna N.M. (2018). Comparison of sprint interval and endurance training in team sport athletes. J. Strength Cond. Res..

[21] Engel F.A., Ackermann A., Chtourou H., Sperlich B. (2018). High-intensity interval training performed by young athletes: A systematic review and meta-analysis. Front. Physiol..

[22] Di Credico A., Gaggi G., Ghinassi B., Mascherini G., Petri C., Di Giminiani R., Di Baldassarre A., Izzicupo P. (2020). The influence of maturity status on anthropometric profile and body composition of youth goalkeepers. Int. J. Environ. Res. Public Health.

[23] Laursen P.B., Jenkins D.G. (2002). The scientific basis for high-intensity interval training: Optimising training programmes and maximising performance in highly trained endurance athletes. Sports Med..

[24] Gibala M.J., Little J.P., MacDonald M.J., Hawley J.A. (2012). Physiological adaptations to low-volume, high-intensity interval training in health and disease. J. Physiol..

[25] Di Credico A., Izzicupo P., Gaggi G., Di Baldassarre A., Ghinassi B. (2020). Effect of physical exercise on the release of microparticles with angiogenic potential. Appl. Sci..

[26] Gibala M.J., McGee S., Garnham A.P., Howlett K., Snow R.J., Hargreaves M. (2009). Brief intense interval exercise activates AMPK and p38 MAPK signaling and increases the expression of PGC-1α in human skeletal muscle. J. Appl. Physiol..

[27] Bangsbo J., Iaia F.M., Krustrup P. (2008). The yo-yo intermittent recovery test. Sports Med..

[28] Buchheit M. (2008). The 30-15 intermittent fitness test: Accuracy for individualizing interval training of young intermittent sport players. J. Strength Cond. Res..

[29] Dupont G., Defontaine M., Bosquet L., Blondel N., Moalla W., Berthoin S. (2010). Yo-Yo intermittent recovery test versus the Université de Montréal track test: Relation with a high-intensity intermittent exercise. J. Sci. Med. Sport.

[30] Iv J.J.D., Gruber A.H. (2019). Quantifying exposure to running for meaningful insights into running-related injuries. BMJ Open Sport Exerc. Med..

[31] Izzicupo P., Di Blasio A., Di Credico A., Gaggi G., Vamvakis A., Napolitano G., Ricci F., Gallina S., Ghinassi B., Di Baldassarre A. (2020). The length and number of sedentary bouts predict fibrinogen levels in postmenopausal women. Int. J. Environ. Res. Public Health.

[32] Aadland E., Ylvisåker E. (2015). Reliability of the actigraph GT3X+ accelerometer in adults under free-living conditions. PLoS ONE.

[33] Clark B.K., Healy G.N., Winkler E.A.H., Gardiner P.A., Sugiyama T., Dunstan D.W., Matthews C.E., Owen N. (2011). Relationship of television time with accelerometer-derived sedentary time. Med. Sci. Sports Exerc..

[34] Colby M.J., Dawson B., Heasman J., Rogalski B., Gabbett T.J. (2014). Accelerometer and GPS-derived running loads and injury risk in elite australian footballers. J. Strength Cond. Res..

[35] Hennessy L., Jeffreys I. (2018). The current use of GPS, its potential, and limitations in soccer. Strength Cond. J..

[36] Gómez-Carmona C.D., Bastida-Castillo A., Ibáñez S.J., Pino-Ortega J. (2020). Accelerometry as a method for external workload monitoring in invasion team sports. A systematic review. PLoS ONE.

[37] Staunton C., Wundersitz D., Gordon B., Kingsley M. (2017). Construct validity of accelerometry-derived force to quantify basketball movement patterns. Int. J. Sports Med..

[38] Staunton C., Wundersitz D., Gordon B., Kingsley M. (2018). Accelerometry-derived relative exercise intensities in elite women’s basketball. Int. J. Sports Med..

[39] Boyd L.J., Ball K., Aughey R.J. (2011). The reliability of MinimaxX accelerometers for measuring physical activity in Australian football. Int. J. Sports Physiol. Perform..

[40] Fulkerson B. (1995). Machine learning, neural and statistical classification. Technometrics.

[41] Cust E.E., Sweeting A.J., Ball K., Robertson S. (2019). Machine and deep learning for sport-specific movement recognition: A systematic review of model development and performance. J. Sports Sci..

[42] Bassett D.R., Rowlands A., Trost S. (2012). Calibration and validation of wearable monitors. Med. Sci. Sports Exerc..

[43] Farrahi V., Niemela M., Tjurin P., Kangas M., Korpelainen R., Jamsa T. (2020). Evaluating and enhancing the generalization performance of machine learning models for physical activity intensity prediction from raw acceleration data. IEEE J. Biomed. Health Inform..

[44] Kerr J., Carlson J., Godbole S., Cadmus-Bertram L., Bellettiere J., Hartman S. (2018). Improving hip-worn accelerometer estimates of sitting using machine learning methods. Med. Sci. Sports Exerc..

[45] Winter E.M., Maughan R.J. (2009). Requirements for ethics approvals. J. Sports Sci..

[46] Cormack S., Smith R.L., Mooney M.M., Young W.B., O’Brien B. (2014). Accelerometer load as a measure of activity profile in different standards of netball match play. Int. J. Sports Physiol. Perform..

[47] Nicolella D.P., Torres-Ronda L., Saylor K.J., Schelling X. (2018). Validity and reliability of an accelerometer-based player tracking device. PLoS ONE.

[48] Schober P., Boer C., Schwarte L.A. (2018). Correlation coefficients: Appropriate Use and Interpretation. Anesth. Analg..

[49] Vehtari A., Gelman A., Gabry J. (2017). Practical bayesian model evaluation using leave-one-out cross-validation and WAIC. Stat. Comput..

[50] Grünbichler J., Federolf P., Gatterer H. (2020). Workload efficiency as a new tool to describe external and internal competitive match load of a professional soccer team: A descriptive study on the relationship between pre-game training loads and relative match load. Eur. J. Sport Sci..

[51] Rago V., Brito J., Figueiredo P., Costa J., Barreira D., Krustrup P., Rebelo A. (2019). Methods to collect and interpret external training load using microtechnology incorporating GPS in professional football: A systematic review. Res. Sports Med..

[52] Aughey R. (2011). Applications of GPS technologies to field sports. Int. J. Sports Physiol. Perform..

[53] Laursen P., Buchheit M. (2019). Science and Application of High-Intensity Interval Training: Solutions to the Programming Puzzle.

[54] Buchheit M., Laursen P.B. (2013). High-intensity interval training, solutions to the programming puzzle: Part II: Anaerobic energy, neuromuscular load and practical applications. Sports Med..

[55] LeCun Y., Bengio Y., Hinton G. (2015). Deep learning. Nature.

[56] Filippini C., Cardone D., Perpetuini D., Chiarelli A., Gualdi G., Amerio P., Merla A. (2021). Convolutional neural networks for differential diagnosis of raynaud’s phenomenon based on hands thermal patterns. Appl. Sci..

